# Aging of Urban Traffic Fine Particles Modulates Toxicity Mechanisms and Oxidative Stress Responses

**DOI:** 10.1021/acs.est.6c06323

**Published:** 2026-10-08

**Authors:** Michal Pardo, Noga Kozer, Carolina Molina, Yanfang Chen, Haim Barr, Galit Cohen, Damianos Pavlidis, Christos Kaltsonoudis, Katerina Seitanidi, Georgia A. Argyropoulou, Christina N. Vasilakopoulou, Christina Christopoulou, Angeliki Matrali, Mingchen Wei, Andre S. H. Prevot, David M. Bell, Spyros N. Pandis, Athanasios Nenes, Yinon Rudich

**Affiliations:** † Department of Earth and Planetary Sciences, Faculty of Chemistry, Weizmann Institute of Science, 234 Herzl Street, P.O.Box 26, Rehovot 7610001, Israel; ‡ Maurice and Vivienne Wohl Institute for Drug Discovery, Nancy and Stephen Grand Israel National Center for Personalized Medicine, Weizmann Institute of Science, Rehovot 7610001, Israel; § Institute of Chemical Engineering Sciences (FORTH/ICE-HT), Patras 26504, Greece; ∥ PSI Center for Energy and Environmental Sciences, Paul Scherrer Institute (PSI), Villigen 5232, Switzerland; ⊥ Department of Chemical Engineering, 37795 University of Patras, Patras 26504, Greece; # Université Paris Cité and Univ Paris Est Creteil, CNRS, LISA, F-75013 Paris, France; ∇ School of Architecture, Civil & Environmental Engineering, École Polytechnique Fédérale de Lausanne, Lausanne CH-1015, Switzerland; ○ Aix-Marseille Université, Université de Toulon, CNRS, IRD, MIO, 13288 Marseille Cedex 09, France

**Keywords:** Secondary organic aerosol (SOA), Mitochondrial dysfunction, Oxidative potential (OP), Cell Painting, parking garage, signaling mechanisms

## Abstract

Vehicular emissions are an important source of urban air pollution that can age through atmospheric processes, but how their health effects change during atmospheric aging remains poorly understood. To simulate realistic urban emissions, we sampled air in a parking garage where cold-start and urban emissions coexist. Following aging in an oxidation flow reactor (OFR), we studied the cellular and acellular effects of the fresh and aged aerosols. Photochemical aging in the OFR increased secondary organic aerosol (SOA) mass by up to 6.9-fold, the oxygen-to-carbon (O:C) ratio from 0.26 to 0.79, and both the intrinsic oxidative potential (OPm) and the volume-normalized oxidative potential (OPv). In contrast, the peroxide-sensitive DCF activity decreased following aging. Cellular exposure to aged aerosol extracts increased cellular reactive oxygen species (ROS) formation in lung (A549) and liver (HepG2) cells, whereas fresh extracts strongly reduced cellular respiration, particularly in HepG2 cells. Cell painting analysis showed distinct morphological profiles. Exposure to aged extracts showed greater oxidative stress and DNA damage responses than those of exposure to fresh extracts. These findings suggest that atmospheric aging shifts the toxicity of fresh emissions from suppression of mitochondrial respiration toward oxygenated organics that can drive intracellular oxidative stress and DNA damage phenotypes. In conclusion, atmospheric aging of cold-start and urban vehicular emissions contributes to enhanced SOA formation and leads to distinct biological outcomes that depend on the degree of oxidative aging.

## Graphical abstract

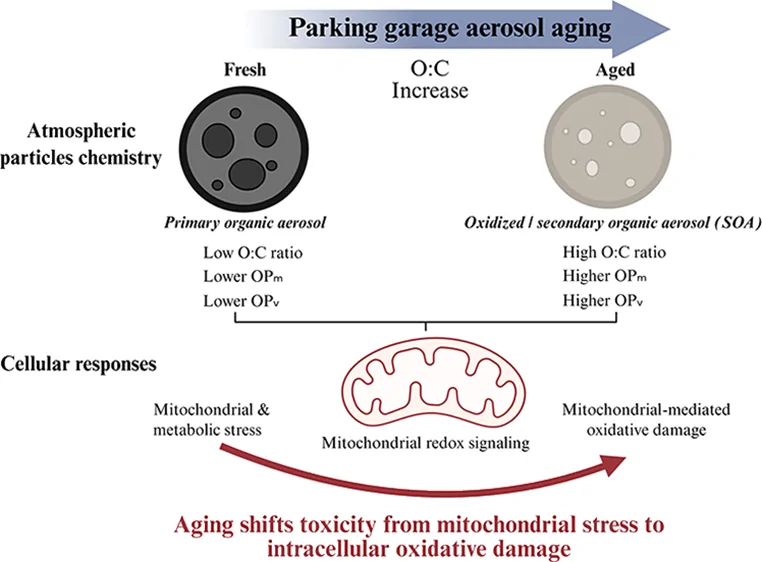

## 1 Introduction

Vehicle emissions are a major source of urban air pollution and are associated with adverse health outcomes.^1,2^ The majority of emissions occur in open air; they can however also occur in semiclosed microenvironments like parking garages.^3–5^ Cold starts, the period of vehicular operation where catalytic converters are not warm enough to optimally reduce emissions, are a significant but understudied source of particles and gas emissions.^6,7^ The contribution of cold starts to urban pollution increases as ambient temperature drops.^8^ Vehicle emissions increase during frequent cold starts, especially in semiclosed environments where vehicles idle and restart.^9^ Incomplete combustion leads to enhanced emissions of volatile (VOCs), intermediate volatility (IVOCs), and semivolatile organic compounds (SVOCs).^6,10,11^ These compounds can be oxidized in the atmosphere, increasing the concentration of secondary organic aerosol (SOA).^12^ This results in an aerosol mixture containing freshly emitted and partially aged aerosols, which are not well represented in ambient roadside or urban measurements.

Atmospheric aging alters the chemical composition of the primary combustion aerosols. Fresh traffic emissions contain high levels of soot (black carbon), which is often coated by SOA during atmospheric aging, changing the physicochemical properties and the potential toxicity of the emitted particles.^13–15^ Fresh emissions from combustion processes are also enriched in elemental carbon (EC), metals, polycyclic aromatic hydrocarbons (PAH), quinones, and other redox-active species.^6^ Aging can transform organic compounds into more oxygenated organic species (e.g., peroxides or reactive SOA) and thus change their biological effects.^16^

A key mechanism by which combustion emissions exert their toxicity is through the induction of oxidative stress.^17,18^ Oxidative stress is an imbalance between the production of reactive oxygen species (ROS) and the capacity of cells to detoxify them with antioxidants.^19^ Excessive ROS can damage cellular lipids, proteins, and DNA, which lead to impaired cell viability and function. Oxidative stress is damaging to metabolically active organs, such as the liver and lungs, which are primary sites of detoxification and gas exchange, respectively. The lungs are directly exposed to ambient particles. Small (ultrafine) particles and soluble components can translocate into the bloodstream and reach remote organs such as the liver, where they may induce inflammation, oxidative damage, and metabolic disruption.^19^ Therefore, vehicular emissions can affect not only respiratory conditions but also liver pathologies, including inflammation and lipid dysregulation.^16,20^

Acellular OP assays quantify the redox activity of particulate matter (PM) and link aerosol chemistry to potential cellular responses.^1,21,22^ Atmospheric aging can modify particle composition and redox activity, but the ability of OP assay responses to predict cellular oxidative stress mechanisms is limited.^23,24^ Moreover, mitochondrial respiration is a sensitive target of particle-induced oxidative stress and ROS generation,^19^ but its response to fresh and aged vehicle emissions remains poorly characterized. These limitations highlight the need for cellular assays that can capture pathway-specific responses to complex environmental mixtures. Cell painting is a target-agonist high-content imaging method that uses fluorescent staining and automated image analysis to detect changes in cell morphology after chemical exposure.^25,26^ This approach can identify cellular responses associated with oxidative stress, DNA damage, and mitochondrial dysfunction^25–27^ and provide a complementary strategy to acellular or single-end point assays.

The biological implications of exposure to freshly emitted particles versus atmospherically aged aerosols remain largely underexplored. A few laboratory studies have used a dynamometer setup to measure the effect of fresh and aged emissions on lung cells.^28^ The EU-funded EASVOLEE project (http://www.easvolee.eu/) funded a campaign in an indoor parking garage that sampled vehicle-emitted aerosols representing an urban environment with frequent cold-start emissions, and examined how aging affects the oxidative effects using several complementary novel assays. Specifically, acellular OP measurements were combined with intracellular ROS production and oxygen consumption rate (OCR) in human lung (A549) and liver (HepG2) cells, together with Cell Painting analysis to reveal the underlying cellular mechanisms. These cell models were chosen to represent the lung as the primary site of exposure and the liver as a key organ for metabolism, allowing us to examine both local and systemic cellular responses. Therefore, we assess the differences between “Fresh” and “Aged” aerosols and their impact on oxidative and mitochondrial stress.

## 2 Materials and Methods

### 2.1 Sampling Site

The atmospheric processes were studied in the largest indoor parking garage facility of the Veso Mare shopping center in Patras, Greece, from the end of November to the end of December 2024. The parking garage has 250 parking spaces, and more than 20,000 cars visited the parking garage during the study. The parking garage was ventilated with outdoor air and, therefore, was not a completely isolated environment.

To investigate secondary aerosol production and to simulate atmospheric aging, air sampled from the parking garage was introduced into an oxidation flow reactor (OFR),^29^ operating under continuous flow conditions.^30^ The hydroxyl radical (OH) exposure, which determines the degree of oxidation, was controlled by varying the intensity of UV light in the OFR. “Fresh” aerosols refer to particles sampled directly from the parking garage, while “aged” particles indicate air that passed through the OFR and correspond to ∼0.5–2 atmospheric oxidation days. The instruments alternately measured fresh air and OFR-aged air, switching every ∼21 min. A total of 21 fresh and 21 aged samples were collected. Organic aerosol (OA) mass concentrations ranged from ∼7 μg m^–3^ in fresh aerosols to ∼34–130 μg m^–3^ in aged aerosols.

### 2.2 Chemical Characteristics of Fresh and Aged Emissions from a Parking Garage

Online and offline instruments were used to characterize aerosol and gas-phase composition in the parking garage and downstream of the OFR; aerosol number size distributions were measured using a scanning mobility particle sizer (SMPS #1, TSI Inc., model 3034, Shoreview, MN, USA; 10–490 nm), a second SMPS (TSI Inc., classifier model 3080, DMA model 3081, CPC model 3775, Shoreview, MN, USA; 14–710 nm), and an optical particle sizer (OPS, TSI Inc., model 3330, Shoreview, MN, USA).

Aerosol chemical composition was characterized by a high-resolution time-of-flight aerosol mass spectrometer (Aerodyne Research Inc., model HR-ToF-AMS, Billerica, MA, USA), a proton-transfer reaction time-of-flight mass spectrometer (PTR-MS) (IONICON Analytik GmbH, model PTR-TOF 6000, Innsbruck, Austria) equipped with a chemical analysis of aerosol online (CHARON) inlet (IONICON Analytik GmbH, Innsbruck, Austria), an Aethalometer (Magee Scientific, model AE33, Berkeley, CA, USA), an extended-range soot particle photometer (Droplet Measurement Technologies, model SP2-XR, Longmont, CO, USA), and an electrical low-pressure impactor (Dekati Ltd., Kangasala, Finland). The gas-phase composition was characterized using PTR-MS together with continuous monitoring of NO, NO_2_, CO, CO_2_, O_3_, and SO_2_. Fresh aerosol samples were collected on quartz filters using a PM_2.5_ high-volume sampler (HVS, AMS Analitica, Pesaro, Italy) and operated at a flow rate of 250 L min^–1^ for an average sampling duration of 23.9 h. Aged aerosol samples were collected downstream of the OFR. The PM_2.5_ filter samples were used for offline chemical, oxidative potential, and biological analyses. In contrast, the PM_1_ concentrations reported in the results were obtained from the online aerosol measurements (AMS and Aethalometer). Gas-phase samples from the parking garage were collected on Tenax TA sorbent tubes for offline analysis. Elemental and organic carbon concentrations were determined with a thermal optical carbon analyzer (Sunset Laboratory Inc., model 5L, Tigard, OR, USA), following the EUSAAR2 protocol.^31^

### 2.3 Oxidative Potential (OP) Assays

OP of the fresh and aged particles was measured using a dithiothreitol (DTT) assay (OP^DTT^).^32–34^ Sections of filter (fresh or aged) were extracted in ultrapure water (18.2 MΩ cm^–1^, TOC below 2 ppb) using an ultrasonic bath (Elma S50, 37 kHz, 150 W) for 30 min, then filtered with poly­(tetrafluoroethylene) (PTFE) filters (0.45 μm) to remove filter fibers and insoluble particles before the OP and biological assays, and analyzed using a semiautomated system comprising syringe pumps (Cadent 3, Norgren), a liquid waveguide capillary cell (LWCC; LWCC-M100, WPI), and an online UV–vis spectrometer (Ocean Optics). Blank filters were extracted and filtered in the same way and used as controls for the OP assays. Extracts were mixed with potassium phosphate buffer (pH: 7.4, KH_2_PO_4_ and K_2_HPO_4_) and DTT (100 μM in the reaction vial) and incubated at 37 °C with constant agitation (ThermoMixer C, Eppendorf); aliquots from the incubated reaction vial were then mixed with trichloroacetic acid (TCA), 5,5′-dithiobis (2-nitrobenzoic acid) (DTNB), and Tris buffer. The decrease in DTT concentration was recorded considering the formation of the product thionitrobenzoic acid (TNB), detected spectrophotometrically at 412 nm. For quality control, 9,10-phenanthrenequinone (PQN) and deionized water were used. Furthermore, after preparing fresh solutions, a DTT calibration curve was generated to quantify DTT consumption. OP normalized to particle mass (OP_m_^DTT^; nmol DTT min^–1^ μg^–1^) was calculated from the estimated PM_2.5_ carbonaceous species mass as (OA/OC)_AMS_ × OC + EC (Table S1), where (OA/OC)_AMS_ is the OA-to-OC ratio taken from the AMS measurements, and OC is the organic carbon measured with the thermal optical carbon analyzer.^35^ OP_m_^DTT^ represents the intrinsic oxidative capacity of the particles, and OP normalized to volume (OP_v_^DTT^; nmol DTT min^–1^ m^–3^) represents the exposure burden.

OP^DCF^ was measured by 2′,7′-dichlorodihydrofluorescein diacetate (H_2_DCF), with horseradish peroxidase (HRP).^32^ The reactions were performed in black 96-well plates containing environmental extract, 20 μM DCF, and HRP at 0.25 U mL^–1^. Plates were incubated at 37 °C protected from light, and fluorescence was measured kinetically (Ex 485 nm/Em 530 nm) every 1 min for 30 min using a microplate reader. Standard curves with hydrogen peroxide (H_2_O_2_) were prepared freshly before each experiment (0–4 μM), and fluorescence was used to quantify sample OP. The DTT assay is more specific to redox-active organic species (e.g., quinones) and transition metals,^1,18,32^ while the DCF assay is more specific to peroxides and ROS in aqueous environments.

### 2.4 Cell Culture and Exposures

Human lung carcinogenic cell line A549 (ATCC CCL-185) and human epithelial liver cell line HepG2 (ATCC HB-8065) were used for all exposure experiments and were grown as previously described.^36,37^ Briefly, A549 and HepG2 were cultured in RPMI and DMEM (Gibco, Thermo Fisher Scientific, Waltham, MA, US), respectively, supplemented with 10% fetal bovine serum (FBS) and 5 μg/mL penicillin/streptomycin (Biological Industries) and maintained at 37 °C in a humidified atmosphere containing 95% air and 5% CO_2_.

Cell painting analysis was performed separately using U2OS cells, as described below.

The cells (passages 2–30, with consistent passage numbers in each experiment) were exposed to water-soluble extracts from the parking garage samples in serum-free medium with salts/glucose; the medium comprised 50 mM 4-(2-hydroxyethyl)-1-piperazineethanesulfonic acid (HEPES), 100 mM NaCl, 5 mM KCl, 2 mM CaCl_2_, and 5 mM glucose (pH 7.2 prior to use to maintain osmolarity). The cells were exposed to vehicle extracts at 500 μg/mL and to blank extracts (underwent the same procedures as the extracts with water, served as controls). Each well in a 24-well plate received 0.5 mL of the extract solution, corresponding to a total mass of 250 μg per well. Given the surface area of 1.9 cm^2^ per well, the resulting exposure doses were approximately 132 μg/cm^2^, calculated from the extract concentration and volume. Preliminary tests (data not shown) showed no significant responses at lower concentrations. The highest concentration did not affect cell viability; therefore, the highest concentration available across all samples was used. Cellular ROS was measured after 5 h of exposure, whereas cellular respiration (OCR) was monitored continuously for up to 96 h, and the Cell Painting assay was conducted after 24 h of exposure. For each treatment, 21 independent biological samples were analyzed, and each sample was measured in three technical replicates.

### 2.5 Intracellular ROS

Total intracellular reactive oxygen species (ROS) were evaluated by flow cytometry (Amnis CellStream Flow Cytometer, Luminex, US)^36^ using a 25 μM H_2_DCF (Thermo Fisher Scientific) probe, which is reactive to hydrogen peroxide, lipid hydroperoxide, and hydroxyl radicals, with low reactivity to superoxide anions. Five thousand cells were measured with Ex/Em wavelengths of 495/529 nm. Unstained cells were used as the negative control, and cells exposed to 100 μM H_2_O_2_ were used as the positive control and were used to determine the gating settings.

### 2.6 Real-Time Detection of OCR

Real-time detection of the level of the oxygen consumption rate (OCR) was assessed by the Resipher instrument (Lucid Scientific Inc., Atlanta, GA, USA).^38^ Briefly, cells were seeded in a 96-well plate (Falcon), and the sensing lids were placed after the cells were treated with parking garage extracts. The rate of oxygen consumption was calculated continuously for up to 96 h at the end, and the OCR was normalized to the number of cells per well by using PI staining.

### 2.7 Cell Painting

Cell Painting analysis was performed using U2OS cells (human osteosarcoma cells). U2OS cells were used because of their suitability for morphological profiling and their prevalent use in Cell Painting and Mechanism of Action (MoA) reference libraries.^39,40^ The cells were cultured in DMEM (Sartorius, Göttingen, GE), supplemented with 10% heat-inactivated FBS, 2 mM l-glutamine, and 100 units penicillin/streptomycin (Biological Industries, Beit HaEmek, IL) at 37 °C in a humidified atmosphere containing 95% air and 5% CO_2_. The cells were seeded on 384-well plates containing the samples and controls (Greiner #781091) at a 2500 cells/well density. Cell density was optimized to range from 50% to 80% confluent in control cells at the time of imaging. Nine technical replicates were included for each compound in 3 different plates. The compounds were assigned randomly to cancel well position effects. After 24 h of incubation with the samples, the plate was stained with five fluorescent dyes using Phenovue Cell Painting Kit (Revvity, Waltham, MA, USA cat# Rev-Ping12) according to the manufacturer’s instructions and imaged by ImageXpress Micro Confocal High-Content Imaging System (Molecular Device, San Jose, CA, USA). Nine images/well were imaged using 20× objective air lens (Nikon, Ph1 S Plan Fluor ELWD) with the following filters: DAPI (4′,6-diamidino-2-phenylindole) (Ex: 377/50 nm, Em: 447/60), FITC (fluorescein isothiocyanate) (Ex:475/34 nm, Em: 520/35), TRITC (Ex: 531/40 nm, Em: 593/40), TexasRed (Ex: 560/32 nm, Em: 624/40), and CY5 (Ex: 631/28 nm, Em: 692/40). All liquid dispensation, compound treatment, and imaging steps were conducted on an automated platform designed specifically for cell microscopy screens. Acquired images were analyzed by CellProfilerTM software (Broad Institute, Cambridge, MA, USA) to generate 1000 features from every well.

### 2.8 Bioinformatics Analysis

Cell Painting images were analyzed in CellProfiler v4 to perform segmentation of nuclei and cell bodies and to extract single-cell features.^41^ For each cell, we measured a wide range of morphological features across all imaging channels, including intensity, texture, size, shape, and spatial relationships between cellular compartments. To reduce plate-to-plate and spatial effects, all features were normalized on a per-plate basis using 60 negative control wells (dimethyl sulfoxide, DMSO) arranged in a grid across each plate.

To summarize high-dimensional phenotypes and facilitate visualization, well-level profiles were projected into a lower-dimensional space by using principal component analysis (PCA). Similarity between profiles was quantified using (generalized) Mahalanobis distance, computed for all profile pairs using a covariance matrix estimated from the negative control (DMSO) profiles, thereby accounting for the correlation structure among features. The resulting distance matrix was subjected to hierarchical clustering, and dendrograms were generated to identify groups of compounds and PCMs with similar morphological signatures.

### 2.9 Statistical Analysis

Results are expressed as mean ± standard deviation (SD). Differences between two independent groups were assessed using an unpaired Welch’s two-tailed *t* test. For multiple comparisons, differences were analyzed using two-way analysis of variance (ANOVA) to assess the effects of treatment and time, followed by Tukey’s honestly significant difference (HSD) post hoc test for multiple comparisons. Differences were considered statistically significant at *P* < 0.05. Statistical analyses and graph generation were performed using GraphPad Prism version 10.6.1 (GraphPad Software, La Jolla, CA, USA).

## 3 Results

### 3.1 Chemical Characteristics of Fresh and Aged Aerosol from the Parking Garage

Gas and aerosol phase measurements were performed in the parking garage. The average PM_1_ concentration measured by AMS and aethalometer was 19.9 ± 17.5 μg m^–3^ (*n* = 21), with black carbon accounting for 5.3 ± 4.0 μg m^–3^, organic aerosol for 13 μg m^–3^ (Table 1), and the remaining mass consisting of inorganic aerosol components. The fresh parking garage air was exposed to three distinct OH levels (by varying the UV light intensity in the OFR) that correspond to approximately 0.5, 1, and 2 days of equivalent midlatitude atmospheric photochemical aging. Following photochemical aging, OA substantially increased by 4.6 times for 0.5 days of aging (OH1), 5.8 times for 1 day (OH2), and 6.9 times for 2 days of OH aging (OH3). This demonstrates strong SOA formation under oxidative conditions. Changes in oxidation state, as indicated by oxygen-to-carbon (O:C) ratios, showed that the fresh aerosol had a low O:C ratio (0.26 ± 0.12), consistent with a mixture of primary vehicle and wood-burning emissions.^42^ The aged aerosol had a higher O:C ratio (OH1, 0.65 ± 0.09; OH2, 0.68 ± 0.10; OH3, 0.79 ± 0.14), indicating that aging produced more oxygenated organic aerosol (Table 1). This increase in oxidation was accompanied by the formation of numerous oxygenated particle-phase SOA components (C_*x*_H_*y*_O_*z*_), including ions such as C_4_H_5_O_2_^+^, C_5_H_7_O_2_^+^, C_6_H_7_O_3_^+^, and C_7_H_9_O_4_^+^, detected in the aged aerosol (Table S2). The O:C values of the aged particles are within the range generally reported for atmospherically aged organic aerosols.^15,43^

**Table 1:** Summary of Fresh and Aged Aerosol Properties^a^

	Fresh		Aged	
		OH_1_	OH_2_	OH_3_
PM_1_ (μg m^–3^)	19.9 ± 17.5	—	—	—
Black Carbon (μg m^–3^)	5.31 ± 4.0	—	—	—
OA increase	—	4.8	5.8	6.9
O:C ratio	0.26 ± 0.12	0.65 ± 0.09	0.68 ± 0.1	0.79 ± 0.14

a OH_1_–OH_3_ correspond to increasing OH exposure levels equivalent to 0.5, 1, and 2 days of atmospheric aging, respectively.

The parking garage was ventilated with outdoor air according to the regulations. Source apportionment measurements were applied to distinguish vehicle emissions from other pollution sources affecting the measurements, using Positive Matrix Factorization (PMF) on the AMS organic aerosol spectra. During winter, biomass burning outside the parking garage influenced the aerosol levels inside, contributing an average of ∼35% of the OA mass in the parking facility and up to ∼50% in the ambient environment, especially during nighttime. This contribution was present as background in both fresh and aged extracts.

### 3.2 OP of Fresh and Aged Emission Extracts from the Parking Garage

To further characterize the extracts, OP was analyzed using the DTT and DCF assays. Fresh aerosol extracts showed mass-normalized OP ( (OP_m_^DTT^), representing the intrinsic oxidative capacity of the particles, of approximately 14 ± 5.9 pmol DTT min^–1^ μg^–1^, and the aged aerosol extracts showed statistically significantly higher values (21 ± 7.6 pmol DTT min^–1^ μg^–1^ Welch’s two-tailed *t* test, *P* = 0.0001), (Figure 1A). Aged aerosols’ volume-normalized OP (OP_v_^DTT^), representing the exposure burden, was higher (1.68 ± 0.58 nmol DTT min^–1^ m^–3^) compared with the fresh aerosols (0.55 ± 0.22 nmol DTT min^–1^ m^–3^) (Figure 1B). The increase in OP_v_^DTT^ reflects both the higher intrinsic OP and the higher aerosol mass produced by SOA formation. Furthermore, the OP was also evaluated using the DCF (OP^DCF^) assay, which showed higher values for the fresh aerosols (6.5 ± 4 arbitrary units, A.U) compared with the aged samples (1.2 ± 0.7 A.U), closer to the detection baseline (Figure 1C). The OP^DCF^ assay is sensitive to the presence of peroxides and ROS in the aqueous solution, suggesting that fresh aerosols have a higher capacity to generate ROS, while aging reduces this activity. Together, these findings indicate that atmospheric aging increases OP measured by the DTT assay and decreases peroxide oxidative activity measured by the DCF assay.

**Figure 1:**
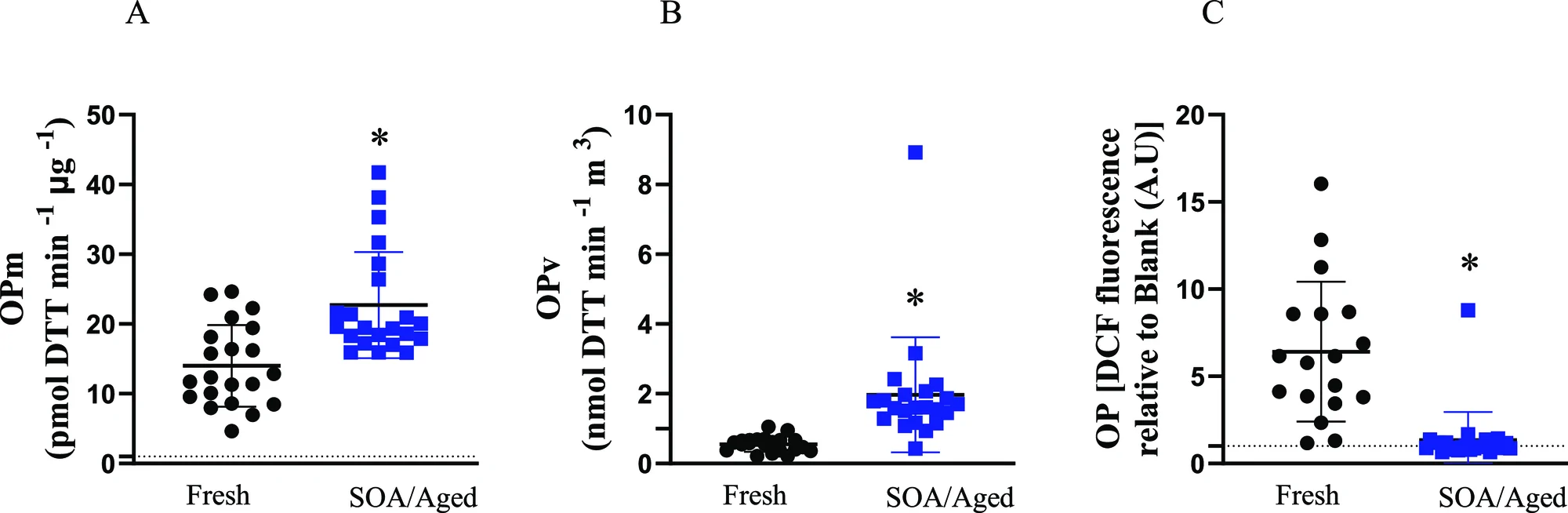
Oxidative potential (OP) of fresh and aged vehicle emission extracts from the parking garage: (A) OP_m_^DTT^, measured as dithiothreitol (DTT) consumption normalized to mass (pmol DTT min^–1^ μg^–1^), (B) OP_v_^DTT^, measured as DTT consumption normalized to sampled air volume (nmol DTT min^–1^ m^–3^). (C) OP^DCF^ assessed by DCF fluorescence and expressed relative to blank extracts. Hydrogen peroxide (H_2_O_2_) standard curves were prepared freshly before each experiment to quantify the OP. Each symbol represents an individual sample; horizontal lines indicate mean ± SD. Dashed lines denote assay detection limits or background levels. Statistical analysis was performed using Welch’s two-tailed unpaired *t* test. * Indicates a statistically significant difference at *P* < 0.05.

### 3.3 Cellular ROS Production and Cellular Respiration in Lung and Liver Cells Exposed to Parking Garage Extracts

To assess the biological impacts of exposure to emission water extracts collected in the parking garage, cells were exposed to fresh and aged extracts. Exposure to aged aerosol extracts resulted in higher cellular ROS levels compared to fresh extracts, as indicated by increased DCF fluorescence in A549 (Figure 2A) and HepG2 cells (Figure 2B), demonstrating enhanced cellular oxidative stress with aging. In contrast, the results of the OCR measurements (Figure 2C and 2D) showed that fresh extracts reduced cellular respiration compared to the blank in A549 and HepG2 cells. Aged aerosol extracts reduced cellular respiration, indicating bioenergetic stress in both HepG2 and A549 cells, with a weaker response in the lung cells (A549 cells). However, the suppression was more pronounced following exposure to the fresh extracts, and the aged extracts had little to no inhibitory effect on the level of OCR compared to the blank. This indicates that although aging increases oxidative stress, fresh emissions may have a greater impact on mitochondrial respiration and metabolic alteration.

**Figure 2:**
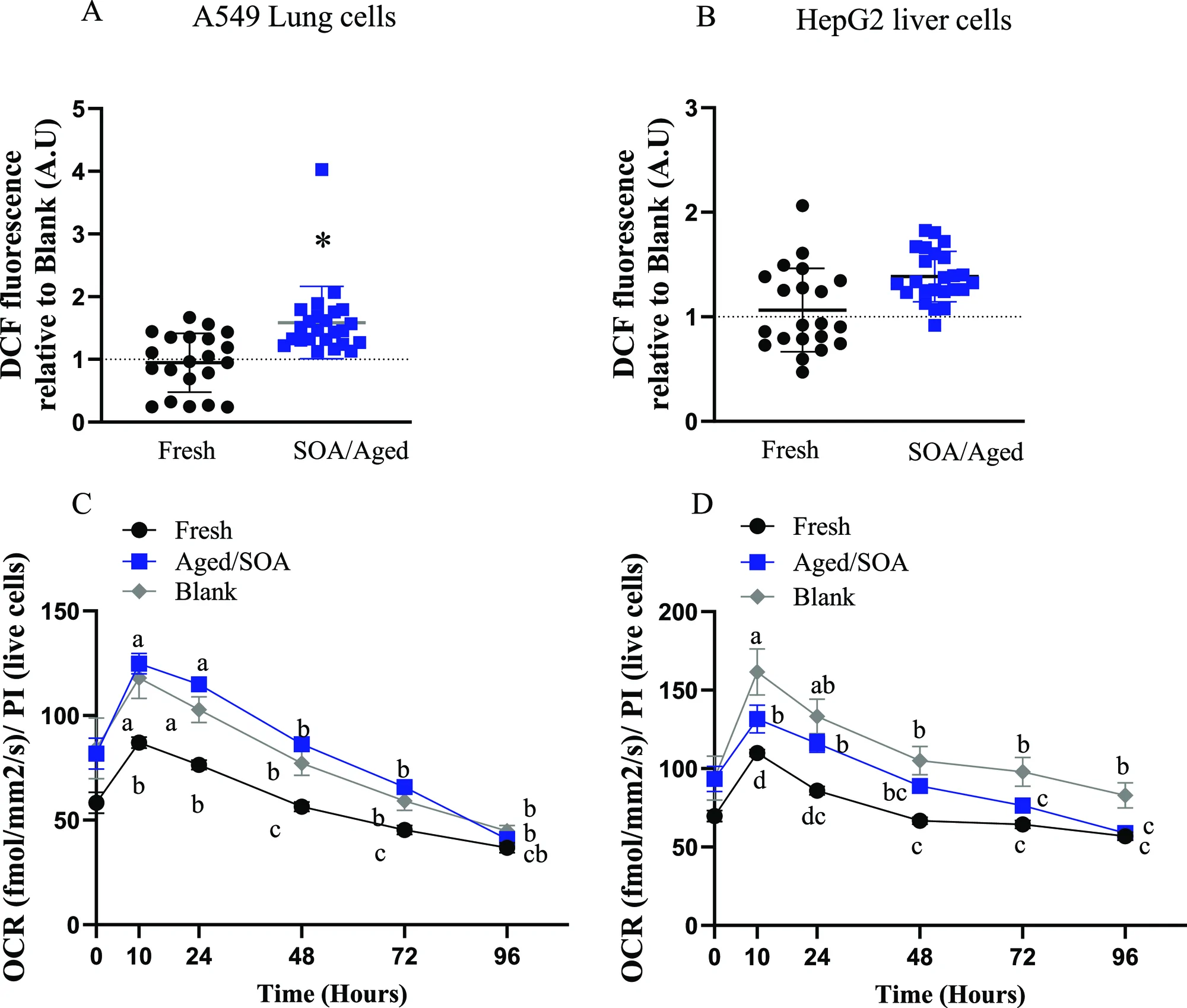
Cellular ROS production and cellular respiration in lung and liver cells exposed to parking garage extracts. A549 lung cells and HepG2 liver cells were exposed to fresh and aged extract from the parking garage for 5 h at a concentration of 132 μg/cm^2^, as indicated in the Materials and Methods section. (A) Cellular ROS production of A549 lung epithelial cells, measured by DCF fluorescence using flow cytometry and expressed relative to blank controls. Pairwise comparisons between two groups were evaluated using a Welch’s two-tailed unpaired *t* test (*P* < 0.05). (B) Cellular ROS in HepG2 liver cells. (C) Oxygen consumption rate (OCR) in A549 lung cells measured over time using the Resipher instrument, reflecting changes in mitochondrial respiration. (D) OCR in HepG2 liver cells. For panels C and D, statistical analysis was performed using two-way ANOVA (treatment × time), followed by post hoc multiple-comparison testing; different letters indicate statistically significant differences between groups at *p* < 0.05. Data are shown as mean ± SD (*n* = 21 biological samples, each measured in triplicate).

### 3.4 Cell Painting Mechanism of Action (MoA) after Exposure to Fresh and Aged Parking Garage Extracts

Cell painting MoA classification shows different phenotypic signatures for blank, fresh, and aged parking garage extracts (Figure 3A–C). Blank samples (Figure 3A) show 100% similarity to cells exposed to DMSO, which are used as control. Exposure to fresh extracts (Figure 3B) shows small changes with respect to the control phenotype, with 85% of the cells classified as the control (DMSO). The other 15% of the cells were similar to the assigned classes (Table 2), to oxidative stress/redox cycling (5%), to oxidative stress/complex mixture response (5%), and to protein degradation (5%). Aged extracts (Figure 3C) produced a stronger phenotypic response. 65% of the cells were similar to DMSO, and 35% were similar to classes associated with cellular damage pathways, oxidative stress/redox cycling (10%), oxidative stress/complex mixture (10%), DNA replication stress (10%), and protein degradation (5%). Together, these profiles indicate that aged extracts trigger more pronounced cellular damage than fresh samples, involving both oxidative stress and DNA-associated toxicity pathways.

**Figure 3:**
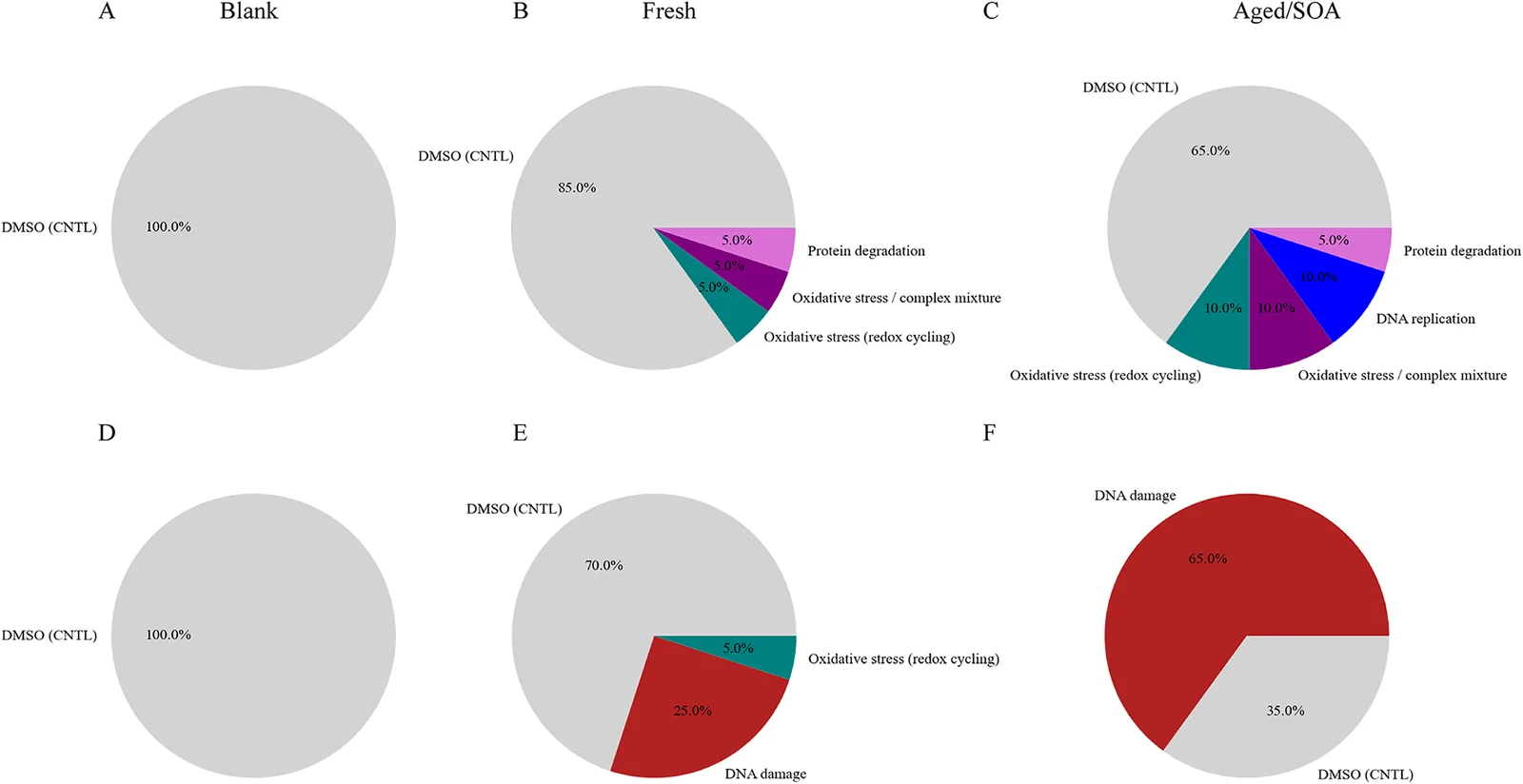
Cell painting mechanism of action (MoA) after exposure to fresh and aged parking lot extracts. Pie charts summarize the distribution of dominant cell painting MoA identified after exposure to fresh and aged parking garage extracts, compared with blank controls. Cell painting MoA for (A) blank controls, (B) fresh samples, and (C) aged samples. CY5 channel MoA for (D) blank, (E) fresh samples, and (F) aged samples. The relative fraction of samples is determined based on similarity to the reference compound signatures found in Table 2. Gray segments represent DMSO controls. Blank samples show DMSO behavior, confirming assay specificity. Data are shown as mean ± SD (*n* = 21 biological samples, each measured in duplicate).

**Table 2:** Reference Compounds Used for Cell Painting Mechanism of Action Classification and Categories

MOA category	Compounds	Concentration (μM)
Negative control	DMSO	1
Microtubule destabilizers	Colchicine	3
	Nocodazole	1
	Vincristine Sulfate	3
Microtubule stabilizers	Paclitaxel (Taxol)	3
	Docetaxel trihydrate	3
	Epothilone B	1
DNA damage	Mitomycin C	3
	PLATINOL (cisplatin)	40
	Etoposide	10
DNA replication stress	floxuridine, mitoxantrone	30
		10
Protein synthesis inhibitors	Anisomycin	3
	Cycloheximide	5
	Emetine dihydrochloride	1
Protein degradation	MG-132, MG-101 (ALLN)	1
		3
Kinase inhibitors	PD-169316 (p38 MAP kinase inhibitor PD), Staurosporine	10
		5–10
Actin disruptors	Cytochalasin D	1
Mitochondrial uncoupler	FCCP (Carbonyl cyanide-*p*-trifluoromethoxyphenylhydrazone)	7
Oxidative stress/redox cycling	H_2_O_2_, 1,4-NQ (1,4-Naphthoquinone), 9,10-PQ (9,10-Phenanthrenequinone)	0.1
		1.5
		0.5
^a^Complex environmental mixtures	water-soluble wood tar (WT), NIST1650B (Diesel particulate matter), NIST1649 (Urban dust)	0.83 μg/mL
		40 μg/mL
		40 μg/mL

a Complex environmental mixtures do not have a defined molecular weight; therefore, concentrations are reported as μg/mL rather than molar units.

The CY5 (Mitotracker, mitochondrial) channel showed the most consistent differences between fresh and aged extracts; therefore, we further analyzed this channel separately. Blank samples (Figure 3D) showed 100% similarity to the DMSO control. Exposure to fresh extracts (Figure 3E) resulted in 70% of the cells being classified as the control (DMSO). The other 30% of the cells matched oxidative stress/redox cycling (5%) and DNA damage (25%). In the aged extracts (Figure 3F), 70% of the cells matched DNA damage and 30% to DMSO. These results indicate that mitochondria are a major target of the extracts and that fresh extracts induce a mild, reversible oxidative stress response, whereas aged extracts induce stronger DNA damage responses, including oxidative stress and protein degradation, that could lead to permanent damage.

To compare how aged and fresh extracts influence CY5 phenotypes, we calculated a toxicity single score per sample that summarizes its deviation from the control in the CY5 channel. The CY5 signal is captured by two complementary analysis modules: Transfluor (vesicle-associated/punctate structures) and MWCS (MultiWave Cell Scoring) (mitochondrial intensity and texture features). Higher scores indicate profiles more similar to the aged extracts, while lower scores indicate profiles closer to the fresh extracts. Across both modules (Figure S1), fresh and aged samples showed overlapping PCA distributions. The scores showed positive and negative values in both groups, and the mean score was slightly higher for aged extracts in both the Transfluor (Figure S1A–C) and MWCS modules (Figure S1D–F), although the variability within groups was large. Thus, the aged extracts tend to produce stronger CY5 morphological responses, but the magnitude of the effect is mixed, and the phenotypic distributions of fresh and aged samples remain partially overlapping.

## 4 Discussion

We find that aging from cold-start vehicular emissions increased the SOA mass, O:C ratios, and the intrinsic redox activity of the particles (OP_m_^DTT^). Aged extracts induced stronger intracellular ROS levels, and morphological features by Cell Painting were associated with oxidative DNA damage and stress response pathways. Fresh extracts inhibited mitochondrial respiration, consistent with reversible metabolic pathway disruption. Aging makes particles more biologically active, possibly by forming compounds that can readily enter and distribute within the cells and induce permanent oxidative damage responses, whereas fresh emissions temporarily impair mitochondrial function and cellular energy metabolism (Table 3).

**Table 3:** Summary of the Chemical and Cellular Response between Fresh and Aged Aerosol Extracts

	Fresh	Aged
Chemistry	Primary organic aerosol	Oxidized organic aerosol
OP-DTT (OPv)	OP-DTT (OPv)	OP-DTT (OPv)
OP-DCF	More peroxide in extract	Less peroxide in extract
Cellular ROS	Lower intracellular ROS	Higher intracellular ROS
Cellular respiration (OCR)	Strong OCR suppression	Mild OCR suppression
Biological phenotype (Cell Painting)	Metabolic stress dominant	Oxidative/DNA stress dominant

Water-soluble extracts from the fresh and aged particles collected in the parking garage showed clear chemical differences following oxidative aging. The aged extracts contained more oxygenated organic material than the fresh extracts, consistent with cold-start exhaust that undergoes atmospheric processing. For example, OFR aging of 0.5–2 equiv days increased the O:C ratio, resulting in the formation of CHO/CHON species and highly oxidized aromatic compounds.^15^ Furthermore, modern vehicle exhaust, including from the cold-start phase, produced SOA during chamber aging, accompanied by an increase in O:C and the formation of nitrate- and sulfate-containing particles.^43^ These studies support the notion that the composition changes in aged parking garage extracts result from the oxidative aging of primary emissions and SOA formation.

To link the chemical changes to the related biological exposures, the *in vitro* doses correspond to PM_2.5_ concentrations measured in closed-parking garages or other microenvironments (∼50–200 μg/m^3^).^44,45^ At these concentrations, an adult that inhales about 0.5–1 m^3^/h would be exposed to several hundred micrograms of PM_2.5_ over a few hours. This mass is consistent with realistic short-term exposures in a high-pollution urban microenvironment.^45^ Previous studies have shown that PM_2.5_ from different urban sources induces considerable changes in cellular oxidative stress levels at equal mass concentrations, indicating that particle mass alone does not determine biological relevance.^46^

Cellular measurements such as intracellular ROS formation and OCR capture downstream biological responses, including mitochondrial and metabolic effects. During atmospheric aging, soot particles are coated with SOA, and particle-bound PAH or other aromatic compounds can be oxidized, leading to ring-opening reactions and to the formation of more fragmented or oxygenated products.^47^ Depending on the oxidation pathway, these reactions may consume PAHs while forming oxygenated aromatic compounds, including quinones and other oxygenated PAHs, that are capable of redox cycling and contribute to oxidative stress.^48^ Previous studies have shown that atmospheric aging can transform the redox activity of combustion emissions by changing the ratio between primary aromatic compounds and the formation of secondary oxygenated species.^14,49–51^ In the present study, aging increased the concentration of gas-phase carbonyls such as acetone and of oxygenated particle-phase SOA components including ions (e.g., C_4_H_5_O_2_, C_5_H_7_O_2_, C_8_H_7_O_2_, and C_10_H_9_O_3_). These products indicate a complex environmental mixture of oxidized compounds from primary emission (e.g., VOCs, IVOCs, and SVOCs), matching secondary formation processes and chemical aging by multiple emission sources. These processes generate reactive oxygenated species such as peroxides and aldehydes that can enhance cellular ROS formation. The chemical differences between fresh and aged extracts affected cellular respiration (OCR): fresh extracts (less oxidized primary emissions) suppressed mitochondrial respiration, whereas more oxidized aged extracts induced milder effects on the level of cellular ROS but stronger cellular ROS formation.

Each OP assay is sensitive to different chemical species: DTT is sensitive to redox-active organics and transition metals from brake wear and other traffic emissions,^1,18,32^ while DCFH primarily detects peroxides and hydroperoxides. The OP^DTT^ assay showed a significant increase in OP_m_^DTT^ following atmospheric aging, indicating that photochemical processing enhanced the intrinsic OP of the particles on a mass basis. This increase was accompanied by a substantially higher OP_v_^DTT^, reflecting both the enhanced intrinsic OP and the increased aerosol mass produced during SOA formation in the OFR. These findings suggest that atmospheric aging increases the oxidative burden through greater SOA mass and changes in particle chemistry that enhance redox activity measured by DTT. Previous studies have reported that extended oxidative processing can transform or reduce active aromatic or quinone precursors measured by DTT, sometimes resulting in little or no change in OP_m_^DTT^ despite chemical aging.^52–54^ In contrast, our results show a significant increase in OP_m_^DTT^, suggesting that the chemical aging conditions in the parking garage generated additional active oxygenated species that compensated for the loss of reactive precursors. Recent online studies similarly reported increased formation of oxygenated and redox-active species following atmospheric aging that contribute to oxidative potential and ROS activity.^18,23,43^ This may suggest that people exposed to aged air pollution may experience a greater oxidative dose,^52,54^ and that OP_m_ alone does not always reflect the biologically relevant oxidative burden.

To evaluate changes in peroxides, hydroxyl radicals, and lipid hydroperoxides, the acellular OP-DCF assay was also used.^1,32^ Fresh extracts showed higher acellular OP-DCF than aged extracts, which were close to the baseline levels. This is consistent with a recent study showing that fresh SOA generate higher ROS than aged SOA, suggesting that atmospheric aging depletes reactive organic hydroperoxides, and reducing the ROS-generating capacity of aged particles.^55^ Nevertheless, the aged extracts induced stronger cellular ROS in both HepG2 and A549 cells. This suggests that atmospheric aging changes the chemistry of the fresh particles, generating more redox-active oxygenated species that can penetrate the cells and disrupt mitochondrial function. These compounds are not efficiently detected by the acellular assays (OP), but they apparently become reactive once they enter the cells.^56^ Aging increases the content of oxygenated species (a higher O:C ratio) and forms carbonyls, quinones, and nitrated aromatics, which are more polar and soluble^57–59^ and may penetrate and distribute more easily within cells and interact with intracellular organelles. These compounds can generate ROS (superoxide, O_2_^•–^, and H_2_O_2_) by redox cycling, especially quinones.^58^ Together, we suggest that atmospheric aging shifts the aerosol composition toward soluble oxygenated organics that are less detected by OP^DCF^ but become redox-active once they enter the cells. The cellular responses therefore involve other intracellular mechanisms, including antioxidant depletion, enzymatic redox cycling, and mitochondrial redox that are linked to OCR changes.^16,22,32,56^ In particular, the more oxygenated and polar compounds in aged aerosols^23^ may interact more efficiently with intracellular components and overwhelm intracellular antioxidant defenses,^60^ leading to increased ROS formation and DNA damage. Although OP_m_^DTT^ increased in the aged extracts, the stronger increase in intracellular ROS and DNA damage suggests that atmospheric aging modifies not only the magnitude of OP, but also the biological mechanisms through which oxidative stress is generated. Aged compounds may be more effective in oxidizing DNA and lipids than the DTT molecule. In contrast, less oxidized components in fresh emissions may interact with lipid membranes and mitochondrial processes, allowing cellular antioxidants to counteract oxidative stress more efficiently and mainly result in reversible effects on cellular metabolism. This difference in reactivity and interaction with antioxidant systems may help explain the enhanced intracellular ROS formation in the aged extracts. Our findings are different from previous studies of laboratory-generated SOA from single precursors,^13,61^ which reported increased peroxide concentrations and cellular ROS following atmospheric aging.^59,61^ The present study investigated a chemically complex urban aerosol mixture and used different peroxide assays and offline water extracts, which may not preserve short-lived peroxide species. These differences in aerosol composition and methodology can contribute to the different peroxide responses observed after aging.

The Cell Painting assay can capture morphological changes due to exposure to various toxicants across multiple cellular compartments. It is widely used in toxicity screening and biomedical research.^26,27^ Using standard references, it is used to link exposure to specific cellular response pathways.^27^ In this study, aged extracts showed a broader distribution of cell painting categories, including oxidative stress, DNA replication/damage, and protein degradation, whereas fresh samples showed fewer stress-related pathways, consistent with previous cell painting studies.^26^ The mitochondrial (CY5) channel showed noticeable changes between the fresh and aged extracts. Fresh samples aligned with mitochondrial stress and reduced respiratory capacity, in agreement with the OCR suppression, but were less strongly affected compared to the aged extracts. It was previously shown that traffic-related and diesel-derived particles reduce ATP-linked respiration, disrupt mitochondrial cristae structure, and impair electron-transport chain activity, leading to metabolic stress and energy dysregulation.^62,63^ *In vitro* and *in vivo* studies demonstrated that mitochondrial alterations involve changes in mitochondrial DNA content and integrity, as well as altered mitochondrial biogenesis signaling, linking aerosol exposure, mitochondrial dysfunction, and downstream cardiometabolic risk.^56,62–64^ The aged extracts resulted in stronger DNA damage and an oxidative stress response, consistent with their enhanced intracellular ROS activity. These findings are in line with studies showing that oxygenated and redox-active pollutants promote oxidative DNA damage, histone remodeling, and stress-response transcriptional programs that are related to mitochondrial redox signaling.^56,62–65^ The oxidative stress induced by the aged samples probably indicates that the mitochondria act as a signaling organelle.^66^

Despite these differences, some overlaps between fresh and aged samples indicate that atmospheric aging does not shift toxicity toward a single response, but possibly rather reflects the variability among the fresh and aged extracts and the underlying oxidative pathways. Cell Painting results, combined with the biochemical assays, support a mechanistic pathway. Fresh emissions extracts impair mitochondrial function and cellular energy metabolism, whereas the aged extracts tend to induce more oxygenated SOA that induces phenotypes dominated by intracellular oxidative stress and DNA damage pathways. Consistently, atmospheric oxidation increased aerosol oxidation, SOA mass, and the intrinsic acellular redox activity, indicating that chemical aging alters toxicity pathways in addition to increasing redox potency. Fresh emissions strongly suppressed mitochondrial respiration, consistent with hydrophobic redox-active components that disrupt cellular bioenergetics, whereas aged aerosols induced greater intracellular ROS and oxidative stress–associated morphological signatures, including DNA damage phenotypes. Together, these results indicate that atmospheric aging transforms vehicular aerosol toxicity from acute metabolic impairment toward oxidative stress-related cellular damage, highlighting that secondary atmospheric processes modify not only particle composition but also the biological mode of action.

This study has several limitations: our measurements represent exposure only to the water-soluble fractions of the aerosols; therefore, the measured OP and toxicity of the particles do not include insoluble constituents (e.g., metal oxides or hydrophobic organic compounds) and represent the OP and toxicity of the water-soluble fraction. High variability and small sample sizes make it harder to detect small differences in OP_m_^DTT^. In addition, our assays were performed offline on filter extracts and not online. Although offline extracts provide complementary insights, some semivolatile compounds and short-lived reactive species may have been lost during filter sampling, extraction, and sample preparation, whereas online methods detect these reactive intermediates more effectively.^18^ The biological responses were evaluated using *in vitro* cell models under submerged exposure conditions, which may not represent *in vivo* effects such as immune responses or systemic effects, or preserve direct aerosol deposition and volatile or semivolatile particle components.

Furthermore, the exposure doses and extract conditions used in the assays do not fully replicate real-world exposure in closed-parking environments. Occasionally, infiltration of outdoor air influenced the parking garage air composition by introducing emissions from external sources, such as biomass burning, which were not fully separated on some sampling days. Finally, the atmospheric aging conditions applied in this work represent a photochemical processing pathway, whereas in a real garage environment several other processes may also occur, possibly producing different chemical and toxicological outcomes.

Although there are no specific regulatory limits for PM_2.5_ in indoor parking environments,^67^ our results suggest that cold-start emissions and their SOA contribute to the exposure risk. Furthermore, these findings highlight that the chemical evolution of the particles is an important factor to consider for potential biological effects.

## Supplementary Material



## Notes

PM_1_ mass data used for oxidative potential normalization by measurement date; identification of oxygenated particle-phase ions in aged SOA using CHARON–PTR-TOF-MS; and additional mitochondrial morphological feature analysis of the CY5 channel (DOCX)
