# Supplementary material for: Aging of Urban Traffic Fine Particles Modulates Toxicity Mechanisms and Oxidative Stress Responses
Source: Environ Sci Technol. 2026 Oct 8;60(41):29313–24. doi: 10.1021/acs.est.6c06323 (PMC13689821; doi:10.1021/acs.est.6c06323)
Supplement: Supplementary file 1 [file es6c06323_si_001.docx]

Supporting information

**Aging of urban traffic fine particles modulates toxicity mechanisms and oxidative stress responses**

Michal Pardo ^1, *^, Noga Kozer ^2^, Carolina Molina ^3^, Yanfang Chen ^4^, Haim Barr ^2,^ , Galit Cohen ^2^, Damianos Pavlidis ^3,5^, Christos Kaltsonoudis ^3^, Katerina Seitanidi ^3^, Georgia A. Argyropoulou ^3,5^, Christina N. Vasilakopoulou ^3,6^, Christina Christopoulou ^3,5^, Angeliki Matrali ^3,5^, Mingchen Wei ^7,8^, Andre S. H. Prevot ^4^, David M. Bell ^4^, Spyros N. Pandis ^3,5^, Athanasios Nenes ^3,5,7^, and Yinon Rudich ^1, *^

^1^ Department of Earth and Planetary Sciences, Faculty of Chemistry, Weizmann Institute of Science, 234 Herzl Street, P.O.Box 26, Rehovot 7610001, Israel

^2^ Maurice and Vivienne Wohl Institute for Drug Discovery, Nancy and Stephen Grand Israel National Center for Personalized Medicine, Weizmann Institute of Science, Rehovot 7610001, Israel

^3^ Institute of Chemical Engineering Sciences (FORTH/ICE-HT), Patras, 26504, Greece

^4^ PSI Center for Energy and Environmental Sciences, Paul Scherrer Institute (PSI), Villigen, 5232, Switzerland

^5^ Department of Chemical Engineering, University of Patras, Patras, 26504, Greece

^6^ Université Paris Cité and Univ Paris Est Creteil, CNRS, LISA, F-75013 Paris, France

^7^ School of Architecture, Civil & Environmental Engineering, École Polytechnique Fédérale de Lausanne, Lausanne CH-1015, Switzerland

^8^ Aix-Marseille Université, Université de Toulon, CNRS, IRD, MIO, 13288 Marseille Cedex 09, France

* Corresponding authors, [Michal.levin@weizmann.ac.il](mailto:Michal.levin@weizmann.ac.il). [Yinon.rudich@weizmann.ac.il](mailto:Yinon.rudich@weizmann.ac.il)

Table S1. PM₁ mass for OPₘ normalization.

| Date | Sample | OC (µg/cm²) | | EC (µg/cm²) | | OA/OC | PM₁ (µg/cm²) |
| --- | --- | --- | --- | --- | --- | --- | --- |
| 29-Nov | Fresh | 31.16 | 40.75 | | 9.60 | | 1.50 |
| 30-Nov | Fresh | 32.74 | 43.15 | | 10.41 | | 1.54 |
| 1-Dec | Fresh | 27.57 | 42.18 | | 14.61 | | 1.45 |
| 2-Dec | Fresh | 54.23 | 70.48 | | 16.24 | | 1.45 |
| 3-Dec | Fresh | 35.93 | 46.88 | | 10.95 | | 1.48 |
| 4-Dec | Fresh | 55.86 | 69.04 | | 13.18 | | 1.54 |
| 5-Dec | Fresh | 34.95 | 48.09 | | 13.13 | | 1.51 |
| 6-Dec | Fresh | 39.96 | 52.81 | | 12.85 | | 1.51 |
| 7-Dec | Fresh | 36.80 | 49.40 | | 12.61 | | 1.48 |
| 8-Dec | Fresh | 27.86 | 34.91 | | 7.05 | | 1.59 |
| 9-Dec | Fresh | 60.20 | 76.55 | | 16.36 | | 1.47 |
| 10-Dec | Fresh | 50.98 | 63.97 | | 12.98 | | 1.46 |
| 11-Dec | Fresh | 92.98 | 130.20 | | 37.21 | | 1.37 |
| 12-Dec | Fresh | 52.73 | 68.93 | | 16.21 | | 1.48 |
| 13-Dec | Fresh | 54.74 | 71.14 | | 16.41 | | 1.50 |
| 14-Dec | Fresh | 40.17 | 49.24 | | 9.07 | | 1.57 |
| 15-Dec | Fresh | 71.10 | 94.24 | | 23.14 | | 1.50 |
| 16-Dec | Fresh | 106.25 | 178.32 | | 72.07 | | 1.51 |
| 17-Dec | Fresh | 78.38 | 111.63 | | 33.25 | | 1.51 |
| 18-Dec | Fresh | 88.83 | 123.65 | | 34.82 | | 1.55 |
| 19-Dec | Fresh | 73.49 | 93.74 | | 20.25 | | 1.60 |
| 2-Dec | SOA | 32.13 | 33.59 | | 1.46 | | 2.03 |
| 4-Dec | SOA | 26.73 | 29.25 | | 2.52 | | 1.96 |
| 5-Dec | SOA | 17.59 | 19.21 | | 1.34 | | 1.98 |
| 6-Dec | SOA | 25.61 | 27.87 | | 2.26 | | 2.06 |
| 7-Dec | SOA | 29.58 | 32.19 | | 2.61 | | 2.20 |
| 8-Dec | SOA | 16.52 | 18.29 | | 1.77 | | 2.04 |
| 9-Dec | SOA | 15.84 | 17.87 | | 2.02 | | 2.01 |
| 10-Dec | SOA | 21.10 | 23.16 | | 2.06 | | 2.19 |
| 11-Dec | SOA | 24.72 | 27.11 | | 2.40 | | 2.02 |
| 12-Dec | SOA | 9.59 | 12.10 | | 2.50 | | 1.51 |
| 13-Dec | SOA | 19.61 | 22.23 | | 2.62 | | 2.03 |
| 14-Dec | SOA | 8.16 | 8.39 | | 0.23 | | 1.99 |
| 14-Dec | SOA | 25.68 | 26.34 | | 0.66 | | 2.08 |
| 14/12-N | SOA | 4.57 | 5.34 | | 0.77 | | 1.59 |
| 15-Dec | SOA | 21.68 | 25.02 | | 3.34 | | 2.03 |
| 16-Dec | SOA | 25.71 | 28.54 | | 2.83 | | 2.15 |
| 18-Dec | SOA | 21.42 | 24.97 | | 3.55 | | 1.97 |
| 19-Dec | SOA | 23.68 | 25.73 | | 2.05 | | 2.14 |
| 20-Dec | SOA | 25.51 | 27.94 | | 2.43 | | 2.00 |
| 21-Dec | SOA | 23.35 | 24.92 | | 1.57 | | 2.00 |
| 22-Dec | SOA | 28.65 | 31.09 | | 2.44 | | 2.00 |
| 23-Dec | SOA | 25.54 | 28.30 | | 2.76 | | 2.00 |

Note: Organic aerosol (OA) was calculated as (OA/OC) _from AMS_ × OC, and PM₁ mass was estimated as OA + EC.

**Identification of oxygenated particle-phase ions using CHARON–PTR-TOF-MS**

Oxygenated ions were identified during oxidative aging experiments using an oxidative flow reactor (OFR). The parking garage air was sampled and processed in a 16 L OFR to simulate atmospheric aging (equivalent to ~0.5–2 days). The molecular composition of the aged air was analyzed using a proton-transfer-reaction time-of-flight mass spectrometer (PTR-TOF-MS, Ionicon PTR-TOF 6000). This instrument is equipped with the Chemical Analysis of Aerosol On-line (CHARON) inlet, which enables direct measurement of particle-phase organic compounds. Aerosol particles desorbed in the CHARON inlet and subsequently ionized via proton transfer from H₃O⁺ ions in the PTR drift tube (operated at ~2.8 mbar and ~90 Td). High-resolution mass spectra were analyzed using the Ionicon Data Analyzer (IDA) to determine the elemental composition (CxHyOz⁺) of the ions.^1^ Possible compound assignments were made based on accurate mass and comparison with previously reported oxidation products from aromatic SOA formation studies.^2^

Table S2. Oxygenated ions detected in aged particle-phase SOA during OFR aging

| Ion | m/z | Possible compound / class | Oxygen atoms | |
| --- | --- | --- | --- | --- |
| C_4_H_5_O_2_⁺ | 85 | butendial | 2 |  |
| C_5_H_7_O_2_⁺ | 99 | methylfuranone / 4-oxo-2-pentenal / methyl betendial | 2 |  |
| C_5_H_5_O_2_⁺ | 97 | furfural | 2 |  |
| C_6_H_9_O_2_⁺ | 113 | dimethyl-furanone / methyl-4-oxo-2-pentenal | 2 |  |
| C_6_H_7_O_3_⁺ | 127 | dimethyl-furandione / trihydroxybenzenes | 3 |  |
| C_5_H_7_O_3_⁺ | 115 | 4-oxo-2-pentenoic acid | 3 |  |
| C_7_H_9_O_3_⁺ | 141 | oxygenated aromatic products | 3 |  |
| C_6_H_7_O_4_⁺ | 143 | muconic acid / dioxo-hexenoic acid | 4 |  |
| C_7_H_9_O_4_⁺ | 157 | tetrahydroxy-toluene | 4 |  |

Note: Data from CHARON–PTR-MS analysis of aged SOA

**Mitochondria morphological features analysis (CY5 channel)**

CY5 images were analyzed based on profiling frameworks.^3-5^ Mitochondria images (CY5 channel) were analyzed for their morphological features using a the Transfluor and MWCS (Multi Wavelength Cell Scoring) MetaXpress modules. Transfluor module was designed to detect and measure mitochondria vesicles while MWCS detected and measured the mitochondria area and intensity.

To quantify differences between fresh and aged samples, module-specific CY5 signatures were constructed by selecting features that discriminated between the two groups. For each sample, we calculated an aged score that shows the average change of selected features relative to the controls. Positive values indicate an “Aged” profile and negative values indicate a “Fresh”. Scores were summarized as mean ± standard deviation.^3^

**Fig. S1.** CY5 analysis reveals distinct vesicular and mitochondrial response patterns in fresh and aged samples. (A) PCA of raw Transfluor (vesicle-related) module, each point represents a single sample. Ellipses show the 95% confidence region for each group. Scores were calculated for each sample by averaging the deviations of CY5 features that distinguished fresh and aged groups, relative to Blanks. (B) Transfluor CY5 scores from aged (positive) to fresh (negative). (C) Mean ± SD Transfluor CY5 scores for fresh and aged samples. (D) PCA of raw CY5 MWCS (mitochondrial) features for individual samples. (E) MWCS CY5 scores for individual samples, (F) Mean ± SD MWCS CY5 scores for fresh and aged samples.

**References**

(1) Al-Naiema, I. M.; Stone, E. A. Evaluation of anthropogenic secondary organic aerosol tracers from aromatic hydrocarbons. *Atmos. Chem. Phys.* **2017**, *17* (3), 2053–2065. DOI: 10.5194/acp-17-2053-2017.

(2) Forstner, H. J. L.; Flagan, R. C.; Seinfeld, J. H. Secondary Organic Aerosol from the Photooxidation of Aromatic Hydrocarbons:  Molecular Composition. *Environmental Science & Technology* **1997**, *31* (5), 1345–1358. DOI: 10.1021/es9605376.

(3) Caicedo, J. C.; Cooper, S.; Heigwer, F.; Warchal, S.; Qiu, P.; Molnar, C.; Vasilevich, A. S.; Barry, J. D.; Bansal, H. S.; Kraus, O.; et al. Data-analysis strategies for image-based cell profiling. *Nature Methods* **2017**, *14* (9), 849–863. DOI: 10.1038/nmeth.4397.

(4) Stossi, F.; Singh, P. K.; Marini, M.; Safari, K.; Szafran, A. T.; Rivera Tostado, A.; Candler, C. D.; Mancini, M. G.; Mosa, E. A.; Bolt, M. J.; et al. SPACe: an open-source, single-cell analysis of Cell Painting data. *Nature Communications* **2024**, *15* (1), 10170. DOI: 10.1038/s41467-024-54264-4.

(5) Nyffeler, J.; Willis, C.; Harris, F. R.; Foster, M. J.; Chambers, B.; Culbreth, M.; Brockway, R. E.; Davidson-Fritz, S.; Dawson, D.; Shah, I.; et al. Application of Cell Painting for chemical hazard evaluation in support of screening-level chemical assessments. *Toxicol Appl Pharmacol* **2023**, *468*, 116513. DOI: 10.1016/j.taap.2023.116513.
